# Trends and Projection of the Incidence of Active Pulmonary Tuberculosis in Southwestern China: Age-Period-Cohort Analysis

**DOI:** 10.2196/48015

**Published:** 2023-12-29

**Authors:** Jinou Chen, Yubing Qiu, Wei Wu, Rui Yang, Ling Li, Yunbin Yang, Xing Yang, Lin Xu

**Affiliations:** 1 Division of Tuberculosis Control and Prevention Yunnan Center for Disease Control and Prevention Kunming China

**Keywords:** pulmonary tuberculosis, age-period-cohort modeling, projection, decomposition of disease burden, bayesian age-period-cohort model

## Abstract

**Background:**

The control of pulmonary tuberculosis (PTB) is critical for achieving the vision of World Health Organization’s End TB goal.

**Objective:**

This study analyzes the temporal trends in PTB incidence associated with age, period, and birth cohorts from 2006 to 2020 in Yunnan, China; projects the PTB burden till 2030; and explores the drivers of PTB incidence.

**Methods:**

The aggregated PTB incidence rates between 2005 and 2020 were obtained from the National Notifiable Disease Reporting System. We used the age-period-cohort model to evaluate the age, period, and cohort effects on PTB incidence. We applied the Bayesian age-period-cohort model to project future PTB incidence from 2021 to 2030. We applied the decomposition algorithm to attribute the incidence trends to population aging, population growth, and age-specific changes from 2006 to 2030.

**Results:**

From 2006 to 2020, the PTB incidence in Yunnan was relatively stable, although the absolute number showed an increase. The net drift was –1.56% (95% CI –2.41% to –0.70%). An M-shaped bimodal local drift and a longitudinal age curve were observed. The overall local drift was below zero for most age groups except for the age groups of 15-19 years (2.37%, 95% CI –0.28% to 5.09%) and 50-54 years (0.41%, 95% CI –1.78% to 2.64%). The highest risk of PTB incidence was observed in the age group of 65-69 years, and another peak was observed in the age group of 20-24 years. Downward trends were observed for both period and cohort effects, but the cohort effect trends were uneven. A higher risk was observed for the birth cohorts of 1961-1970 (rate ratio [RR]_1961-1965_=1.10, 95% CI 0.88-1.38; RR_1966-1970_=1.11, 95% CI 0.92-1.37) and 2001-2010 (RR_2001-2005_=0.92, 95% CI 0.63-1.34; RR_2006-2010_=0.84, 95% CI 0.45-1.58) than for the adjacent cohorts. The Bayesian age-period-cohort model projected that PTB incidence will continually increase from 2021 to 2030 and that PTB incidence in 2030 will be 2.28 times higher than that in 2006. The age-specific change was the leading cause for the growing PTB disease burden.

**Conclusions:**

Although there are several levels and measures for PTB control, the disease burden is likely to increase in the future. To bridge the gap of TB-free vision, our study suggests that public health policies be put in place soon, including large-scale active case-finding, priority prevention policies for high-risk older adult and young adult populations, and reduction of possible grandparent-grandchildren transmission patterns.

## Introduction

Tuberculosis (TB) is a chronic communicable disease caused by *Mycobacterium tuberculosis*, which typically affects the pulmonary tissues and is therefore referred to as pulmonary tuberculosis (PTB). This disease can affect other sites as well. The TB epidemic is a major public health challenge. Globally, until the emergence of the COVID-19 pandemic, TB was the leading cause of death from a single infectious agent [1]. The World Health Organization’s End TB strategy and the United Nation’s Sustainable Development Goal to end the TB epidemic by 2035 has ambitious targets of 95% reduction in TB deaths and 90% reduction in TB new case incidence by 2035 globally [2].

China is one of the highest TB burden countries, with 7.4% of the estimated incident cases worldwide in 2022 [1]. Although a downward trend of TB prevalence has been observed in the recent decades [3], a large number of new PTB cases have caused a high disease burden in the country. Therefore, the estimation of the PTB burden in China is critical for achieving the vision of the End TB goal. Several studies [4-6] have reported on the PTB burden trend across time in China. These studies have shown the historical trend of TB incidence, but because those studies failed to differentiate the detailed contributions of age, calendar period, and birth cohort to the overall temporal trends, the effects of the TB control policy before 2005 were hard to evaluate. Additionally, research on prospective trends and projecting the future trends of TB burden is limited. It is necessary to evaluate the localized TB burden in specific regions to improve the control strategy. We addressed these knowledge gaps by examining the age-, period-, and cohort-associated TB incidence in the Yunnan province of China by age-period-cohort (APC) analysis.

The APC approach is a statistical framework for the trend estimation of disease incidence and mortality, and this approach has been extensively applied in cancer epidemiology [7-11], infectious disease modeling [12,13], and chronic disease research [14]. A wide range of research fields are required to understand the temporal changes in disease outcomes attributed to 3 components by applying the APC model. First, the age effects were the changes related to the biological and behavioral changes that affected the aging of individuals, representing the outcomes in terms of different age groups. Second, the period effects were the changes related to public health or social events in particular years; this component affected all age groups and birth cohorts simultaneously in a calendar period. Third, the cohort effects were the outcome changes of birth in the same calendar years, which reflected the specific epidemiological exposure of the different birth cohorts over the life course.

This study demonstrates the effects of age, period, and cohort on the incidence of PTB in the Yunnan province between 2006 and 2020. This study forecasts the PTB incidence in 2030 by the Bayesian APC (BAPC) model. The decomposition analysis attributes the future PTB incidence changes to population aging, population growth, and age-specific reasons. This study will elucidate the various trends of TB incidence in the future and identify the potential drivers of the changes in PTB incidence. To achieve the Sustainable Development Goal in 2035, this study may provide evidence for future control strategies and help in public health decision-making, design of PTB screening, and resource allocation.

## Methods

### Study Sites

Yunnan, a province in the southwest of China, has 16 prefectures and 129 counties. The population of Yunan was 44,499,123 in 2006, consisting of 22,957,243 males and 21,541,880 females [15,16]. By 2020, the population grew to 48,583,533 consisting of 25,204,786 males and 23,378,747 females [17]. The population census presents the population structure shifting and population aging in Yunnan in 2 decades. The ratio of the population younger than 15 years was 25.96%, 20.73%, and 19.57% and that of the population older than 65 years was 6.09%, 11.06%, and 10.75% in 2000, 2010, and 2020, respectively [15-17].

### Ethical Considerations

This study was submitted to the ethics committee of the Yunnan Center for Disease Control and Prevention. This study does not involve human participants and animals. Ethics approval was not required for this study because we did not include any identifiable data of patients’ personal information, including name, identity information, address, and telephone number. This study only reviewed secondary aggregated data at the population level; therefore, waiver of written informed consent was taken (institutional review board exemption: 2023-14).

### Data Source

Active PTB has been notified through the National Notifiable Disease Reporting System (NNDRS) in China since 2004. Thereafter, the Tuberculosis Information Management System was built and applied to complete a standard TB case notification and follow-up in 2005. PTB incidence data between 2005 and 2020 were collected from NNDRS. Notifying PTB cases is considered as mandatory routine work and is enforced by law in China. When a patient with TB visits a health facility and is diagnosed with presumptive or confirmed PTB by physicians, his or her information must be notified to NNDRS by this health facility. Theoretically, NNDRS can capture the data of all the patients with PTB who seek medical advice in a region. After the presumptive cases are diagnosed further, the aggregated clinically diagnosed and laboratory confirmed cases are considered as PTB incidence. To guarantee the incidence data, the NNDRS includes only new PTB cases that have not been treated or reported earlier. This system can manually or automatically mark and delete the duplicate cases by searching and matching identical information of the full names and residential identification card numbers of historically notified cases. Whenever PTB cases are identified as duplicate status, these are excluded from the incidence statistics in NNDRS. The annual aggregated data contain the incidence numbers and incidence rates of PTB, and the data are stratified by the calendar year of notification, age, and sex. The annual population data between 2005 and 2020 were generated from NNDRS. The source of the projected population data was based on the United Nations report on world population prospects [18]. Our research used the Chinese population projection between 2021 and 2030. We generated the Yunnan provincial population projection by a fixed proportion of the provincial population among the Chinese population.

### Statistical Analysis

All statistical analyses were conducted with R software (version 4.0.2; R Core Team). *P* levels <.05 were considered statistically significant for 2-sided tests.

#### APC Model

In this study, we used the APC model to illustrate the age, period, and cohort effects on PTB incidence simultaneously. This approach attempts to resolve and decompose these effects and identify the potential triggers for the varying PTB incidence trends. Due to the perfect linear dependency of the age, period, and cohort variables, the linear effect of the APC model cannot be uniquely estimated, and the difficulty in solving the model parameters is called the APC identification problem [19,20]. The intrinsic estimator was developed based on the decomposition of the matrix singular value, which could fit a stable solution of parameter estimation [21]. The generic form of the APC model can be written as follows: *Y_ijk_* = log(*λ_ijk_*) = *μ* + *α_i_* + *π_j_* + *γ_k_* + *∈_ijk_*

where *Y_ijk_* is the outcome variable to be explained, assumed to follow a quasi-Poisson distribution. The *Y_ijk_* or log(*λ_ijk_*) correspond to the PTB incidence rate for the *i*th age group, *j*th period, and *k*th birth cohort. The *μ* is the intercept, representing the overall average effect. The error *∈_ijk_* is assumed to follow the additive, independent, and identically normal distribution. The *α*, *π*, and *γ* are the log-linear model coefficients corresponding to age, period, and cohort effects, respectively. The usual constraints are the sum of the parameters to zero, presented as ∑*α_i_* = ∑*π_j_* = ∑*γ_k_* = 0.

The APC model requires equal intervals for period, age, and cohort. Therefore, we aggregated the data and then divided each component interval into widths of 5 years. We divided age into 18 age groups (0-4, 5-9, 10-14, …, 80-84, >85), period into 3 periods (2006-2010, 2011-2015, 2016-2020), and cohort into 20 birth cohorts (1921-1925, 1926-1930, …, 2016-2020). A tool developed by the National Cancer Institute was used for the APC model parameters and effects estimation [22]. The net drift, local drifts, longitudinal age curve, period, and cohort rate ratio (RR) were evaluated.

#### BAPC Model

We projected the future incidence of PTB cases from 2021 to 2030 by using the BAPC model. The BAPC model is based on the previously stated population projection. The Bayesian approach divides the deviance into age, period, and cohort effects and treats the unknown parameters as random with an appropriate prior distribution. The second-order random walk smoothing the prior and guaranteeing the second differences in the models were identifiable, which was a random variance assumed to follow an independent mean-zero normal distribution. The BAPC model used the integrated nested Laplace approximation methods to estimate the posterior marginal distributions directly, and then, integrated nested Laplace approximation made extrapolations attributed to age, period, and cohort effects [23,24]. The model fitting was free of Markov Chain Monte Carlo simulations—the reason was to avoid the convergence issues introduced by Markov Chain Monte Carlo sampling that was generally used in the Bayesian approach.

#### Decomposition of the Projected Burden

We further analyzed the triggers for the changes in PTB incidence trends from 2005 to 2030 by the decomposition approach. The decomposition method was developed recently [25]. Briefly, this method attributes changes in PTB incidence to 3 components: population aging, population growth, and age-specific changes between 2005 and each following year to 2030. The method of decomposition has been documented elsewhere, and details are given in Multimedia Appendix 1. The method of decomposition considers the 2-way and 3-way interaction of these 3 components, and decomposition is more robust than other methods [26]. We analyzed the absolute and relative contributions of the 3 components to the change incidence of PTB based on previous BAPC projections. The absolute contribution was the attributed incidence number, while the relative contribution was estimated as the attributed incidence divided by the total incidence in 2006 × 100%. The positive contributions correspond to an increase in PTB incidence, and the negative contributions correspond to a decrease in PTB incidence. The net change was the total PTB incidence comprising these 3 components. The age-specific changes in PTB incidence refer to all the differences in the incidence that cannot be explained by population aging and population growth.

## Results

### Temporal Trends in PTB Incidence

**Figure 1:**
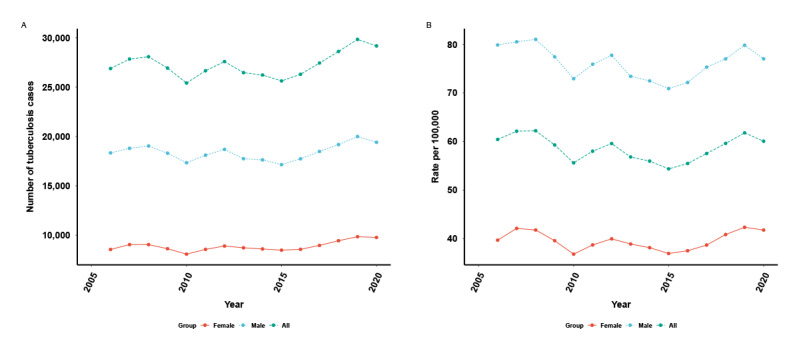
Changes in the number of cases and incidence rate of pulmonary tuberculosis stratified by gender in Yunnan, China, from 2006 to 2020. (A) Number of pulmonary tuberculosis cases stratified by gender. (B) Incidence rate of pulmonary tuberculosis stratified by gender.

**Figure 2:**
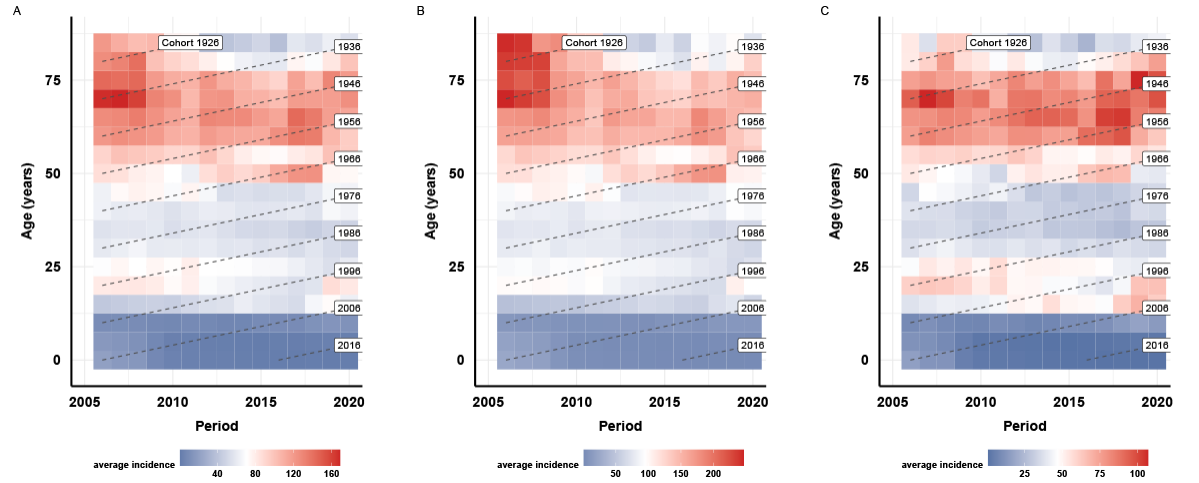
Pulmonary tuberculosis incidence matrix and Lexis diagram by year of diagnosis and age at diagnosis in Yunnan, China, from 2006 to 2020. (A) Incidence matrix of pulmonary tuberculosis. (B) Incidence matrix of pulmonary tuberculosis for males. (C) Incidence matrix of pulmonary tuberculosis for females. Blue represents lower incidence rate, while red represents higher incidence rate. The scales in each panel are different. For each panel, the rows represent the incidence across calendar years for the specific age group, the columns represent the incidence across the specific age group for that calendar year, and the dash lines on the diagonal represent the changes in the incidence for the birth cohort.

In 2006-2020, the annual mean PTB incidence was 58.57 per 100,000; the rate for males (95.71 per 100,000) was higher than that for females (46.77 per 100,000). The sex ratio (male:female ratio) for incidence showed a decline from 2.14 in 2006 to 1.98 in 2020. Between 2006 and 2020, the incidence of PTB increased from 18,336 in 2006 to 19,412 in 2020 for males and from 8552 in 2006 to 9768 in 2020 for females. However, the incidence rate was relatively stable, which was 60.42 per 100,000 in 2006 and 60.06 per 100,000 in 2020 (Figure 1). Across years, the age-standardized incidence rate decreased in most age groups for both males and females, which is presented along each row of the PTB Lexis diagram in Figure 2. A comparison across age groups along each column of the PTB Lexis diagram shows that the group younger than 15 years showed a lower PTB incidence. The highest PTB incidence was observed in the age group of 70-74 years, with the highest PTB incidence among males aged >85 years who were older than the females (age 70-74 years). The diagonals in the Lexis diagram present the different cohorts, overall, and for both sexes. The birth cohorts of 1946-1950, 1951-1955, and 1956-1960 showed higher PTB incidence than other adjacent cohorts, while the birth cohorts of 1981-1985 and 1986-1990 presented higher PTB incidence across periods.

### APC Model Analysis of PTB Incidence

**Figure 3:**
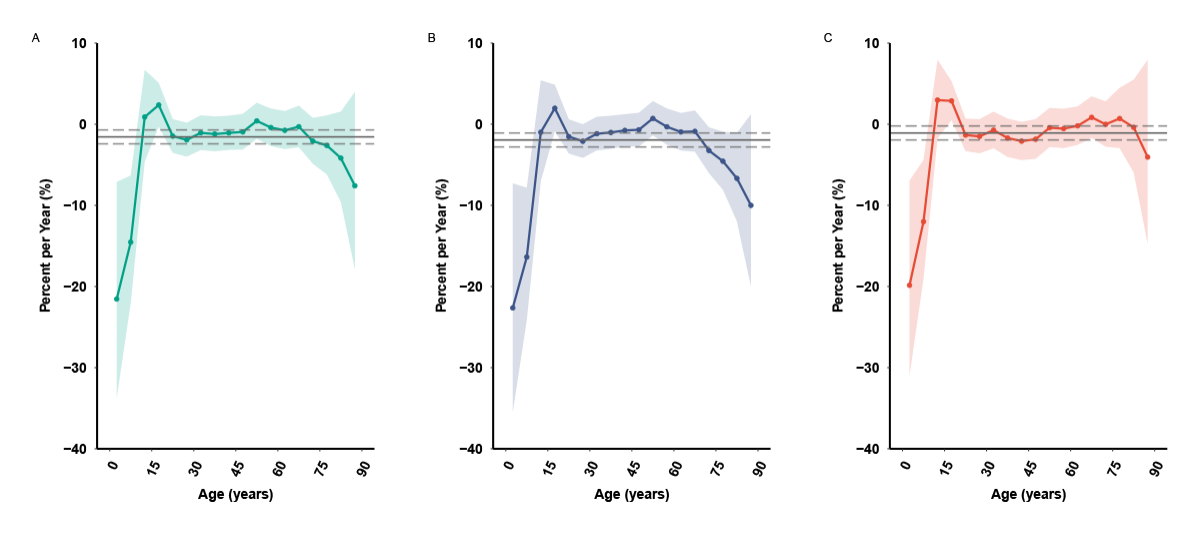
Net drifts and local drifts of pulmonary tuberculosis incidence in Yunnan, China, from 2006 to 2020. (A) Net drifts and local drifts of pulmonary tuberculosis. (B) Net drifts and local drifts of pulmonary tuberculosis for males. (C) Net drifts and local drifts of pulmonary tuberculosis for females. The horizontal grey solid line represents the net drift values, while the grey dash line corresponds to the 95% CIs. The curve with a colored solid line represents the local drift values, and the colored ribbon shows the 95% CIs.

**Figure 4:**
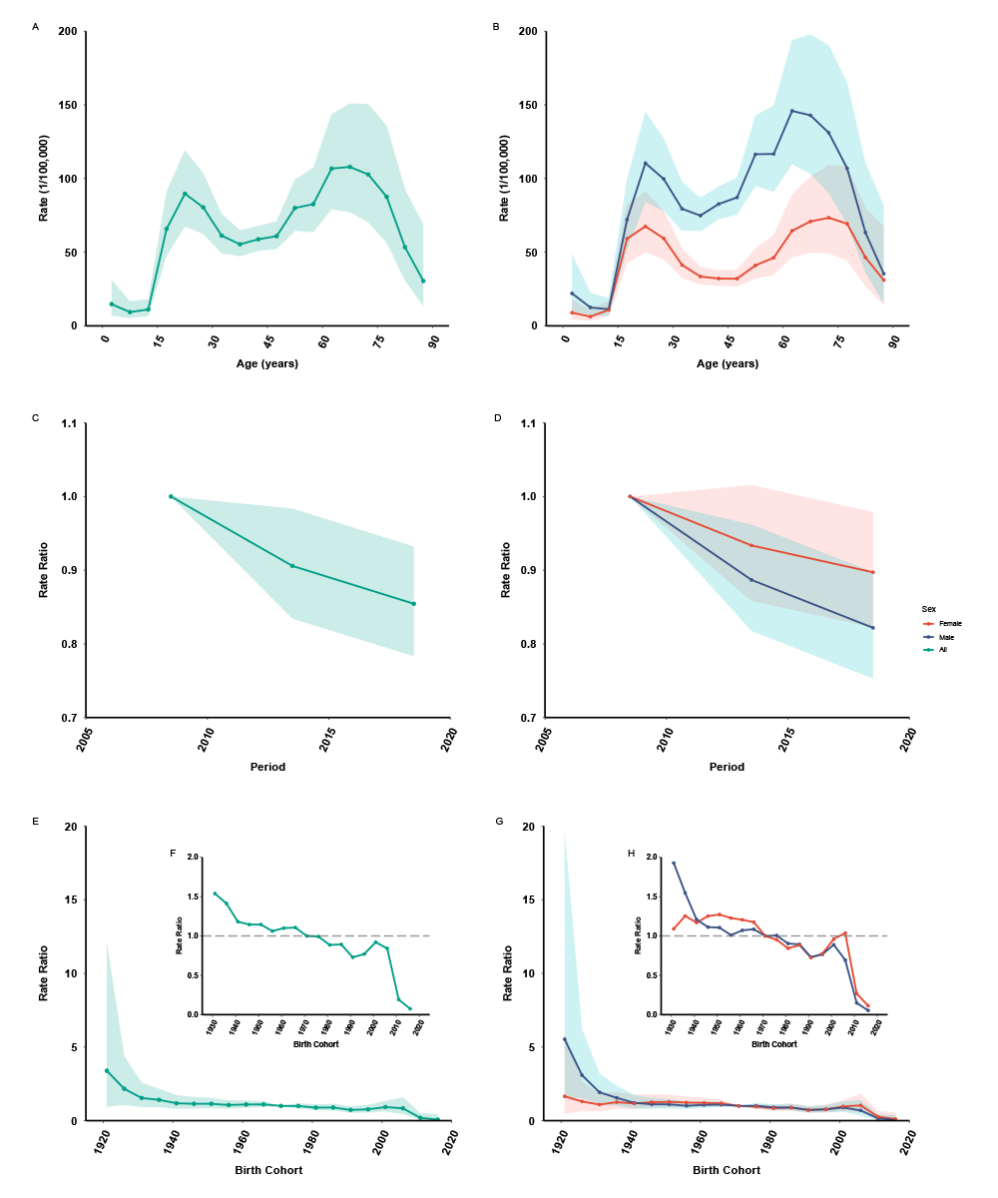
The longitudinal age curve, period, and birth cohort effects curve for pulmonary tuberculosis in Yunnan, China, from 2006 to 2020. (A) Overall longitudinal age curve and the 95% CIs. (B) Longitudinal age curve stratified by gender. (C) Overall period effect and the 95% CIs for pulmonary tuberculosis compared with reference period (2006-2010), adjusted with age and cohort effects. (D) Period effect stratified by gender. (E) Overall cohort effect and the 95% CIs for pulmonary tuberculosis compared with reference cohort (1971-1976), adjusted with age and period effects. (F) Detailed overall cohort effect of birth cohorts between 1931 and 2016. (G) Cohort effect stratified by gender. (H) Detailed cohort effect of birth cohorts between 1931 and 2016, stratified by gender.

The estimated age, period, and cohort effects on PTB incidence are shown in Figure 3 and Figure 4. The net drift presented the overall log-linear annual percentage change of incidence across periods and birth cohorts. The overall net drift was –1.56% (95% CI –2.41% to –0.70%). Differences across sex were observed for males (–1.94%, 95% CI –2.79% to –1.07%) and females (–1.07%, 95% CI –1.94% to –0.21%). PTB incidence among females was lesser than that among males between 2006 and 2020. M-shaped local drift curves were observed, which refer to the difference in the age-specific annual percentage changes. The overall local drift was above zero for 2 age groups, that is, the group aged 15-19 years (local drift 2.37%, 95% CI –0.28% to 5.09%) and the group aged 50-54 years (local drift 0.41%, 95% CI –1.78% to 2.64%). The local drift was below zero for most of the other age groups. The trends of the estimated PTB incidence increased across years for these 2 age groups. Identical effects were observed in both sexes, although the female local drift was above zero for the group aged 65-69 years. The longitudinal age curves presented 2 peaks as well (Figure 4A). For both sexes, after adjusting the period and birth cohort effects, the PTB incidence presented an “up-down-up” trend across ages (Figure 4B). The highest risk of incidence was in the age group of 65-69 years, with another peak around the age groups of 15-19 years, 20-24 years, and 25-29 years.

The period (or cohort) effects were the rate ratios of age-specific rates in each period (or cohort) relative to the reference. Downward trends (RR=0.85, 95% CI 0.78-0.93, 2016-2020 vs 2006-2010) were observed in the period effects for overall and for both sexes. After adjusting for the age and birth cohort effects, a faster decline for the PTB period effect was observed for males (RR=0.82, 95% CI 0.75-0.89, 2016-2020 vs 2006-2010) than for females (RR=0.89, 95% CI 0.82-0.97, 2016-2020 vs 2006-2010, Figure 4C and Figure 4D). The pattern for cohort effects was similar to that for the period effects. The cohort effects continuously declined from the earliest cohort (RR=3.38, 95% CI 0.94-12.08) to the latest cohort (RR=0.07, 95% CI 0.01-0.45). The gender-stratified trends for cohort effects showed a downward trend, with the effects for males declining more rapidly than those for females (Figure 4E and Figure 4G). Furthermore, the downward trend was uneven; the convex cohort effect curve presented a higher RR for the birth cohort 1961-1970 (RR_1961-1965_=1.10, 95% CI 0.88-1.38; RR_1966-1970_=1.11, 95% CI 0.92-1.37) and 2001-2010 (RR_2001-2005_=0.92, 95% CI 0.63-1.34; RR_2006-2010_=0.84, 95% CI 0.45-1.58) than the adjacent cohorts for overall and both sexes (Figure 4F and Figure 4H). Moreover, the net drift, local drifts, period, and cohort effects were all statistically significant (*P*<.05) checked by the Wald *χ*^2^ test (Table S1 of Multimedia Appendix 1).

### BAPC-Projected PTB Incidence

**Figure 5:**
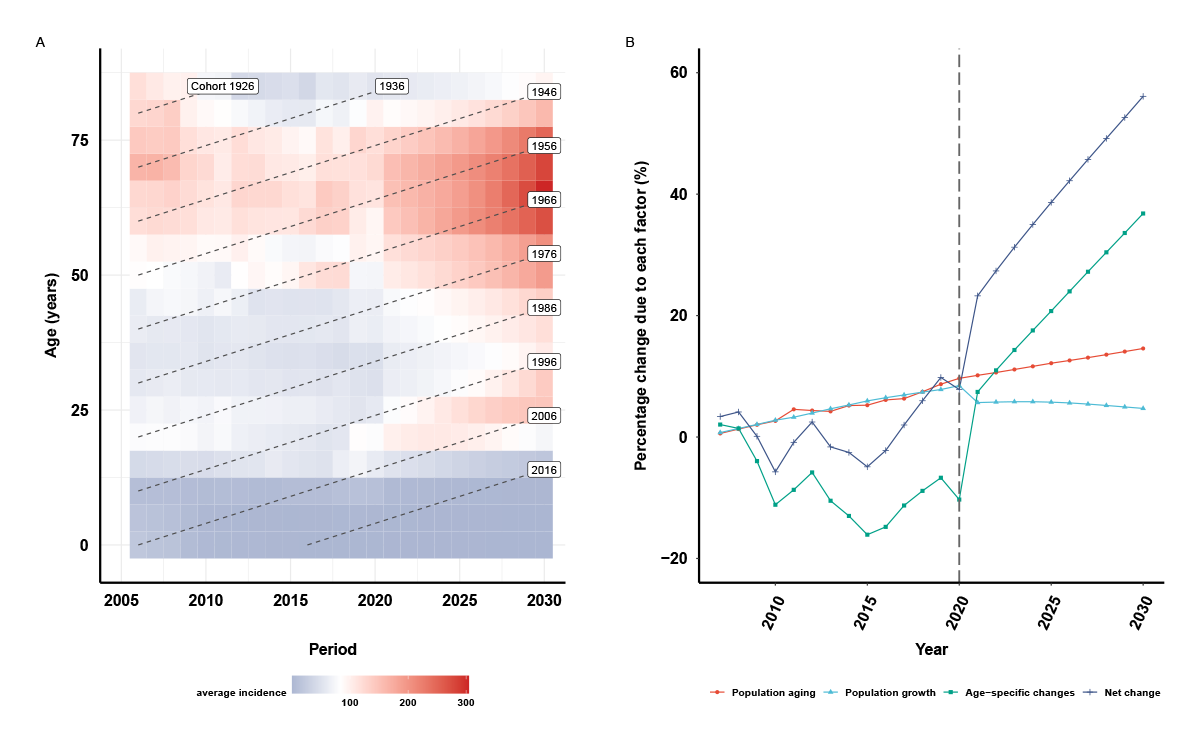
The projected incidence for pulmonary tuberculosis and the decomposition to changes in the pulmonary tuberculosis incidence in Yunnan, China, from 2006 to 2030. (A) The Bayesian age-period-cohort model–projected incidence for pulmonary tuberculosis from 2006 to 2030. (B) Decomposition of the contribution of population aging, population growth, and age-specific changes to the projected incidence of pulmonary tuberculosis from 2006 to 2030.

The BAPC predicted that total PTB incidence would continue to increase, with 61,306 in 2030 in Yunnan, which is 2.28 times that of the year 2006 (Table S2 of Multimedia Appendix 1). However, the projection presented heterogeneity across age groups. Age groups younger than 15 years show a continually decreased PTB incidence, although the older adults and younger adults presented an increasing trend, especially in the birth cohorts of 1961-1970 and 2001-2010 (age range of 60-69 years and 20-29 years, respectively) in 2030 (Figure 5A).

### Decomposition of the PTB Burden

The trigger of PTB incidence from 2006 to 2030 was analyzed by a demographic decomposition. The results showed that age-specific incidence was the primary factor for the incremental trend of PTB incidence (Table 1, Figure 5B). The projection suggested that there are appended 34,396 (56.11%) PTB cases in 2030 compared with the reference year of 2006. The ordered contribution of the 3 factors to the increased trend is the age-specific incidence (36.82% increase from 2006), population aging (14.58% increase from 2006), and population growth (4.71% increase from 2006).

**Table 1:** The projection, decomposition, and contribution of 3 factors to pulmonary tuberculosis incidence estimated by the Bayesian age-period-cohort model from 2007 to 2030 in Yunnan, China.

Year^a^	Total population (N)	Population aging, n (%)	Population growth, n (%)	Age-specific incidence, n (%)	Net changes, n (%)
2007	44,830,030	163 (0.59)	203 (0.73)	571 (2.05)	937 (3.36)
2008	45,139,978	367 (1.31)	393 (1.40)	397 (1.41)	1157 (4.12)
2009	45,430,010	542 (2.01)	558 (2.07)	–1069 (–3.97)	31 (0.12)
2010	45,710,006	675 (2.65)	704 (2.76)	–2839 (–11.15)	–1460 (–5.74)
2011	45,966,362	1216 (4.56)	870 (3.26)	–2321 (–8.70)	–235 (–0.88)
2012	46,310,029	1203 (4.36)	1088 (3.94)	–1610 (–5.83)	682 (2.47)
2013	46,590,006	1117 (4.22)	1231 (4.65)	–2779 (–10.50)	–431 (–1.63)
2014	46,866,000	1360 (5.18)	1385 (5.28)	–3412 (–13)	–667 (–2.54)
2015	47,139,000	1344 (5.24)	1524 (5.94)	–4127 (–16.09)	–1259 (–4.91)
2016	47,418,000	1607 (6.11)	1704 (6.47)	–3897 (–14.80)	–586 (–2.23)
2017	47,710,002	1737 (6.33)	1902 (6.93)	–3096 (–11.28)	543 (1.98)
2018	48,004,978	2132 (7.45)	2116 (7.39)	–2538 (–8.87)	1709 (5.97)
2019	48,299,907	2596 (8.70)	2334 (7.82)	–2003 (–6.72)	2926 (9.81)
2020	48,583,533	2821 (9.66)	2472 (8.47)	–3009 (–10.31)	2284 (7.82)
2021	47,465,047	3561 (10.16)	1988 (5.67)	2602 (7.42)	8152 (23.25)
2022	47,590,518	3934 (10.62)	2131 (5.75)	4067 (10.98)	10,132 (27.35)
2023	47,706,077	4354 (11.12)	2274 (5.81)	5611 (14.33)	12,239 (31.26)
2024	47,792,273	4817 (11.64)	2406 (5.81)	7265 (17.55)	14,488 (35)
2025	47,837,336	5330 (12.15)	2517 (5.74)	9099 (20.75)	16,946 (38.64)
2026	47,850,023	5869 (12.61)	2614 (5.61)	11,168 (23.99)	19,652 (42.21)
2027	47,822,401	6481 (13.07)	2690 (5.43)	13,496 (27.22)	22,667 (45.72)
2028	47,765,525	7182 (13.56)	2750 (5.19)	16,105 (30.42)	26,037 (49.18)
2029	47,697,145	7991 (14.07)	2812 (4.95)	19,080 (33.60)	29,883 (52.62)
2030	47,628,061	8939 (14.58)	2887 (4.71)	22,570 (36.82)	34,396 (56.11)

^a^The year 2006 is the reference.

## Discussion

### Principal Findings

Our study first describes and analyzes the age, period, and cohort effects on PTB incidence in Yunnan, China, between 2006 and 2020 by using the APC model. Then, we predicted the PTB incidence from 2021 to 2030 by using the integrated nested Laplace approximation technique to estimate the BAPC model. Our research further decomposes the drivers of trends and changes in PTB incidence from 2006 to 2030. We found reduction in PTB incidence coupled with negative net drifts in Yunnan between 2006 and 2020. For the age effect, a 2-peak pattern was observed for both local drifts and the longitudinal age curve, which showed that young adults (age 15-29 years) and older adults (age 60-74 years) were high-risk groups for the priority intervention. The APC analysis presented decreased period effects from 2006 to 2020, which suggests the effectiveness of local PTB strategies and other myriad public health implementations in the last few decades. Besides, an exceptional decline presented in cohort effects implies potential transmission patterns. The BAPC predicted a 2.2 times increment in PTB incidence in 2030 compared with that in 2006—the dominant factor that led to the dramatic growth was age-specific changes.

The descriptive analysis showed that from 2006 to 2020, the incidence rate of PTB was stable. However, the incidence increased for both sexes. This inconsistency in the incidence rate and the absolute number of incidence cases is attributed to the population growth in Yunnan in the last few decades [15-17]. The Lexis diagram illustrates a direct landscape of the cross-sectional, longitudinal, and diagonal representation of PTB incidence; the 3-directional depiction explicitly indicated that the PTB incidence among older adults was much higher than that among young adults (age 15-29 years). However, the APC model uncovered the true age pattern of PTB incidence, which was not in line with the Lexis diagram illustration. The APC model presented 2 critical indicators of net drift and local drift. The overall and sex-stratified net drift decreased and was negative in the study period between 2006 to 2020, suggesting that the PTB incidence declined across periods and birth cohorts.

The age effect represents the rate differences among age groups—this may be associated with complex factors such as biological aging, personal health condition, and lifestyle behavior. The age-specific temporal trends were illustrated by longitudinal age curves in this study. The curve showed quite a low incidence in populations younger than 15 years. One possible reason was the national neonatal Bacillus Calmette-Guérin (BCG) vaccination program. The BCG vaccination policy was activated during the 1960s in China. The neonatal BCG vaccination program has been performed nationwide since the 1970s, and the high vaccination coverage has not been interrupted until now. Another possible reason was the difficulty in the diagnosis of pediatric tuberculosis—there were cases of PTB unreported or unnotified due to misdiagnosis. The unreported rate was 58.3%, 20.3%, and 15.3% among populations younger than 15 years, populations aged 16-64 years, and populations older than 65 years, respectively. The risk of unreported tuberculosis in populations younger than 15 years was 7.3 times higher than that in populations older than 65 years [27]. A steep increase was observed in populations older than 15 years—the first incidence peaked in young populations in the age range of 15-29 years. The infection risk in this population may have peaked due to their behavioral changes with large social circles and communication networks. Moreover, the age group of 15-29 years may enjoy a higher degree of freedom and mobility than those younger than 15 years; this correlated with higher chance of exposure to the infectious source of patients with active PTB. The highest peak was observed in older adults aged 65-69 years likely due to immune deficiency diseases, comorbidities, and unhealthy lifestyle behaviors.

The sex differences in the curve may be caused by the different lifestyles of males and females—men have more broader social contacts and networks than women. Besides, men who experienced potential sex-different sociobehavioral risk factors of smoking and alcohol drinking were more likely to develop active PTB, though the difference in incidence between the sexes was not significant in specific age groups. In this study, the M-shaped bimodal curve was observed for PTB incidence rate. The “up-down-up” pattern for the longitudinal age curve was consistent with that reported in other studies in China [5,6]. These consistency trends revealed that young adults (aged 15-29 years) and older adults (aged 60-74 years) were high-risk groups for PTB incidence from a broader perspective.

The local drift distribution was also M-shaped. The definition of local drift is the estimated annual percentage change over time specific to age groups. Our results indicated that PTB incidence increased in populations aged 15-19 years and in those aged 50-54 years across the study period. This finding suggests that instant public health interventions should be prioritized for these high-risk group populations. The PTB burden must significantly decrease in the coming years in China to bridge the gap of the End TB strategy and to achieve the Sustainable Development Goals [28]. Although the active case-finding strategy has been partially implemented as pilot and focused on students and older adults in China [29], the screening policy and preventive therapy specific to high-risk populations should be endorsed as routine work of the national TB program. Case-finding practices in the physical examination screening policy should be implemented routinely for campuses and workplaces, especially for new students and new employees. The screening strategy should consider special sites such as military camps, jails, and nursing homes.

Period effects were modified by macrofactors such as shift in the socioeconomic and medical status, but the period effects on PTB incidence were equal across all age groups and birth cohorts within calendar years. This study presents the continuous decline of the period effect over the past 15 years. The decline is attributed to the elimination strategy, wherein patients with PTB are detected as early as possible and treated with antituberculosis drugs. In the last few decades, the key approach for managing the PTB epidemic in Yunnan has been to control the source of infection. First, Yunnan implemented the directly observed treatment short-course strategy in 2004, which promoted case detection and cured more patients. Second, the surveillance system of NNDRS and Tuberculosis Information Management System were applied simultaneously since 2004, wherein cases were notified and registered by a modernized web-based instant information system. Third, between 2015 and 2017, Yunnan reformed the TB delivery system to the “3-in-1” model. This model referred to the Center of Disease Control and Prevention responsible for comprehensive management, the TB-designated hospital responsible for diagnosis and treatment, and the community-level medical and health care institutions responsible for referral and directly observed treatment short-course management [30]. The tripartite cooperation promoted the accessibility and availability of PTB diagnosis and treatment. Fourth, because there was moderate prevalence of HIV in Yunnan [31], TB/HIV bidirectional screening lowered both the burden of TB and HIV disease, thereby reducing TB transmission among patients with high-risk HIV/AIDS. Fifth, a pilot study in Yunnan suggested that multiple rounds of case-finding enhanced the case-finding strategy, which contributed to a decrease in the TB incidence [29]. Further, the implementation of novel public health approaches such as large-scale application of modern molecular rapid diagnostic techniques of GeneXpert, advancement of the patient care cascade, and development of an advanced information system [32] helped reduce the disease burden in last 2 decades.

The cohort effect known as the generation effect is the varying disease incidence among various generations and birth cohorts associated with specific epidemiological exposure such as wars, famine, baby boom, and disease pandemic. This intergenerational effect could impact the course of life. Our study reveals the earliest birth cohort with the highest RR of PTB and the most recent birth cohort with the lowest RR. This finding is consistent with those reported in other studies in China [5,6,33]. The difference and decrease in the intergenerational effects may be related to the stable socioeconomic status since the establishment of China in 1949. More specifically, the rapid development and continuous economic improvement accompanied by improvement in the quality of life in the past 40 years has led to a reduction in the epidemiological risk exposures for PTB incidence in birth cohorts after 1949.

Another contribution of this study was the finding of higher risks of PTB incidence in the birth cohort of 1961-1970, regardless of the highest and lowest RR cohorts. A possible explanation could be that China endured the great famine from 1959 to 1961, which led to the loss of millions of lives and other health consequences [34-36]. Yunnan, being in the southwestern region of China, has a lower economic status, insufficient food supply, and inadequate transportation in the mountainous rural areas, which led to greater severity in the famine. Biologically, malnutrition during the growth and development stages of children leads to immune system deficiency, consequently causing a higher risk of infection [37,38]. The low nutritional level could be characterized by the lower BMI of the population, which was a definite risk factor for TB incidence [38,39]. Another noteworthy finding was the sudden increasing RR for the birth cohort of 2001-2010. The speculative reason was the intergenerational effect for these 2 birth cohorts. The famine cohort was during 1961-1970, with a supposed average of 20 years for population regeneration [40]—their second-generation offspring was exactly the birth cohort of 2001-2010. Currently, the most popular pattern of bringing up children is that the older adults look after their grandchildren in China. Thus, compared to other age groups, older adults have a significantly increased general contact with those aged 5-10 years [41]. Due to the high probability of PTB incidence among the grandparents, the grandchildren are more likely to contract PTB owing to their close contact. In particular, the speculated grandparent-grandchildren transmission pattern was illustrated by the female cohort curve and expressed as grandmother-granddaughter transmission. Furthermore, the latent tuberculosis infection prevalence increased with age in China, while the risk of active PTB incidence increased synchronously, especially for the generation during the great famine. The hypothetical grandparent-grandchildren transmission pattern would cause more disease burden due to increased latent tuberculosis infection. Regarding the prevention and control of TB, especially in older adult women, the health care policy should consider not only active case-finding but also latent tuberculosis infection surveillance and prevention. Moreover, there was also possibility of nosocomial infections from long-term care facilities and health care systems rather than by community or family member transmission. To sum up, an optimized control policy should involve a comprehensive intervention and screening strategy for older adults.

The BAPC model is particularly useful for predicting future PTB burden, as it involves no parametric assumptions, and it is the only method to achieve sensible projections [24]. Our study shows the striking forecast that PTB incidence will rapidly increase from 2021 to 2030, and by the end of 2030, PTB incidence can increase to 2.28 times higher than that in 2006. The decomposition analysis presents the double PTB incidence attributed to the sequential factors of age-specific changes, population aging, and population growth. The projection and decomposition validated the previous speculation of the grandparent-grandchildren transmission pattern. The famine cohort (birth in 1961-1970) would have the highest PTB incidence in 2030, and their grandchildren cohort (birth in 2001-2010) would have a higher risk than the adjacent cohorts. Although population aging and population growth contributed to only a small portion of PTB incidence, the cohort effect (age-specific changes) can be explained as the leading cause of incidence. Another noteworthy point is the accelerated descent of the Chinese population and fast aging [42-45]. Our findings offer possible explanation for the increasing PTB incidence to be related to the dominant factors of age-specific change and population aging, but the relationship between these factors and their interactions with the projected PTB incidence should be addressed by further evidence.

### Limitations

This study has some limitations. First, the inferences in this study might be limited by ecological fallacy and false logical deduction; these inferences need to be examined by further field and population researches. Second, the intervals of the group for age, period, and cohort were 5 years; although aggregated data reduce potential overdispersion, it could neglect the effect changes in a fine scale. The PTB incidence data were available only of the recent 15 years, which added uncertainty to the long-term analysis and projection. Third, the reported data might underestimate the effects due to underreporting of patients with PTB. Fourth, the uncertainty of the United Nation’s world population prospects projection to future Chinese and Yunnan provincial population sizes and structures and the lack of consideration of migration could unsettle and bias the population-structure–based results of BAPC and decomposition analysis. Considering the Chinese seventh population census conducted in 2020 [17], the effect of population structure shifting should be addressed by further studies. Fifth, the forecast decomposed factors into population aging and growth and age-specific changes. Further research is needed to examine other related factors such as the socioeconomic index, latent tuberculosis infection status, herd immunity, population dietary nutrition level, and the COVID-19 pandemic. Besides, there were residual effects of bias from the APC approach despite the use of the intrinsic estimator methodology. Field investigations are needed to verify the speculated grandparent-grandchildren PTB transmission pattern.

### Conclusion

Our study presents the pattern of PTB incidence in Yunnan, China, and elucidates the underlying causes for the temporal trends by quantifying the age, period, and cohort effects with population-based surveillance data and the macroepidemiological APC model. Although there are several measures and levels for PTB control in China, the PTB burden is likely to increase in the future mainly due to age-specific reasons. The endeavors to achieve the End TB vision in the next decade should include large-scale active case-finding, priority prevention policies for the high-risk older adult and younger populations, and reduction in possible grandparent-grandchildren transmission patterns.

## Appendix

Multimedia Appendix 1 The disease burden decomposition method, parameters of the fitted age-period-cohort model, and projected age-specific number of pulmonary tuberculosis incidence by the Bayesian age-period-cohort model.

## Abbreviations

APC: age-period-cohort
BAPC: Bayesian age-period-cohort
BCG: Bacillus Calmette-Guérin
NNDRS: National Notifiable Disease Reporting System
PTB: pulmonary tuberculosis
RR: rate ratio
TB: tuberculosis

## Data Availability

The data sets analyzed during this study are available from the corresponding author on reasonable request.
