# Supplementary material for: Trends and Projection of the Incidence of Active Pulmonary Tuberculosis in Southwestern China: Age-Period-Cohort Analysis
Source: JMIR Public Health Surveill. 2023 Dec 29;9:e48015. doi: 10.2196/48015 (PMC10787335; doi:10.2196/48015)
Supplement: Multimedia Appendix 1 [file publichealth_v9i1e48015_app1.docx]

**Supplementary Materials**

**The decomposition method**

We take the number of PTB incidence between 2006 and 2030 projected by Bayesian age-period-cohort model, then decomposition the net change of incidence into contribution of three components, population growth, population aging, and age-specific incidence rate.

The age groups were divided into 18 groups using 5-year interval from 0–4 years to 85 plus (we summed the World population Prospects age group of 85–89, 90–94, 95–99, 100+ into age group of 85–).

Let *t_ij_*, *n_ij_*, *m_ij_* and *s_ij_* denote the number of PTB incidence, population size, age-specific incidence rate, and population proportion in the *i*^th^ age group of the year *j*, respectively, (*i* = 1, 2, …, 20; *j* = 1, 2). Let *T*_1_ and *T*_2_, *N*_1_ and *N*_2_, *P*_1_ and *P*_2_ represent the total number of incidence, population size, and the crude rate of incidence in 2006 and 2030 (or the year to be calculated), respectively.

Using *M_p_*, *M_a_* and *M_r_* to represent the main effects of the changes in population size, age structure and incidence rate, and *I_pa_*, *I_pr_*, *I_ar_* and *I_par_* to represent their two-way and three-way interactions, respectively. In the case of 2006 as the reference year, these terms are calculated as follows:

$M_{p}=\sum_{i=1}^{18} {{\left( N_{2}-N_{1} \right)s}_{i1}m}_{i1}$

$M_{a}=\sum_{i=1}^{18} N_{1}\left( s_{i2}-s_{i1} \right)m_{i1}$

$M_{r}=\sum_{i=1}^{18} {N_{1}s}_{i1}\left( m_{i2}-m_{i1} \right)$

$I_{pa}=\sum_{i=1}^{18} \left( N_{2}-N_{1} \right)\left( s_{i2}-s_{i1} \right)m_{i1}$

$I_{pr}=\sum_{i=1}^{18} \left( N_{2}-N_{1} \right)s_{i1}\left( m_{i2}-m_{i1} \right)$

$I_{ar}=\sum_{i=1}^{18} N_{1}\left( s_{i2}-s_{i1} \right)\left( m_{i2}-m_{i1} \right)$

$I_{par}=\sum_{i=1}^{18} \left( N_{2}-N_{1} \right)\left( s_{i2}-s_{i1} \right)\left( m_{i2}-m_{i1} \right)$

Assuming that the interactions are equally distributed, then the contribution of the three factors can be calculated as follows:

$A{=M}_{a}+½I_{ar}+½I_{pa}+⅓I_{par}$

$P{=M}_{p}+½I_{pr}+½I_{pa}+⅓I_{par}$

$R{=M}_{r}+½I_{pr}+½I_{ar}+⅓I_{par}$

Net change = *T_2_ - T_1_*

Here, *A* represents the contribution of population aging, *P* represents the contribution of population growth, *R* represents the contribution of the age-specific incidence rate, and the net change represents total change. The contribution of each factor divided by *T_1_* and multiplied by 100 is the percentage of the respective contribution.

**Table S1.** The Wald’s Chi-square tests for the estimated parameters of the fitted age-period-cohort model

| Null Hypothesis | *χ^2^* | *df.* | *P*-Value |
| --- | --- | --- | --- |
| Overall |  |  |  |
| Net Drift = 0 | 12.57 | 1 | <.01 |
| All Age Deviations = 0 | 185.35 | 16 | <.01 |
| All Period *RR* = 1 | 12.86 | 2 | <.01 |
| All Cohort *RR* = 1 | 37.17 | 19 | .01 |
| All Local Drifts = Net Drift | 31.07 | 18 | .03 |
| Male |  |  |  |
| Net Drift = 0 | 19.24 | 1 | <.01 |
| All Age Deviations = 0 | 176.72 | 16 | <.01 |
| All Period *RR* = 1 | 19.61 | 2 | <.01 |
| All Cohort *RR* = 1 | 45.97 | 19 | <.01 |
| All Local Drifts = Net Drift | 36.89 | 18 | .01 |
| Female |  |  |  |
| Net Drift = 0 | 5.96 | 1 | .01 |
| All Age Deviations = 0 | 228.89 | 16 | <.01 |
| All Period *RR* = 1 | 6.08 | 2 | .05 |
| All Cohort *RR* = 1 | 36.39 | 19 | .01 |
| All Local Drifts = Net Drift | 34.26 | 18 | .01 |

Abbreviation: *RR*=rate ratio

**Table S2.** The projected age-specific number of pulmonary tuberculosis incidence by the Bayesian age-period-cohort model from 2021 to 2030 in Yunnan, China.

| Age group | Calendar year | | | | | | | | | | |
| --- | --- | --- | --- | --- | --- | --- | --- | --- | --- | --- | --- |
|  | 2021 | 2022 | 2023 | 2024 | 2025 | 2026 | 2027 | 2028 | 2029 | 2030 | Row total |
| 0-4 | 25 | 21 | 18 | 16 | 14 | 12 | 11 | 10 | 9 | 8 | 144 |
| 5-9 | 33 | 28 | 24 | 21 | 18 | 16 | 14 | 12 | 11 | 10 | 187 |
| 10-14 | 175 | 155 | 138 | 122 | 107 | 94 | 82 | 72 | 64 | 57 | 1066 |
| 15-19 | 1848 | 1758 | 1642 | 1507 | 1366 | 1233 | 1114 | 1007 | 909 | 818 | 13202 |
| 20-24 | 2937 | 3063 | 3130 | 3142 | 3111 | 3049 | 2944 | 2801 | 2626 | 2439 | 29242 |
| 25-29 | 2595 | 2730 | 2937 | 3186 | 3443 | 3701 | 3919 | 4078 | 4181 | 4244 | 35014 |
| 30-34 | 2712 | 2671 | 2590 | 2533 | 2547 | 2639 | 2818 | 3086 | 3420 | 3788 | 28804 |
| 35-39 | 2698 | 3007 | 3339 | 3618 | 3800 | 3897 | 3896 | 3846 | 3842 | 3961 | 35904 |
| 40-44 | 2301 | 2376 | 2498 | 2683 | 2937 | 3272 | 3704 | 4188 | 4636 | 4992 | 33587 |
| 45-49 | 2845 | 2856 | 2849 | 2853 | 2895 | 2983 | 3128 | 3349 | 3674 | 4123 | 31555 |
| 50-54 | 3572 | 3764 | 3948 | 4117 | 4264 | 4384 | 4468 | 4538 | 4643 | 4831 | 42529 |
| 55-59 | 3345 | 3683 | 4014 | 4332 | 4646 | 4971 | 5318 | 5681 | 6055 | 6432 | 48477 |
| 60-64 | 2855 | 3183 | 3604 | 4095 | 4636 | 5206 | 5821 | 6465 | 7133 | 7850 | 50848 |
| 65-69 | 3209 | 3314 | 3394 | 3531 | 3794 | 4181 | 4738 | 5465 | 6349 | 7381 | 45356 |
| 70-74 | 2261 | 2628 | 3035 | 3422 | 3756 | 4008 | 4200 | 4390 | 4685 | 5187 | 37572 |
| 75-79 | 1032 | 1139 | 1273 | 1449 | 1685 | 1978 | 2350 | 2771 | 3192 | 3587 | 20456 |
| 80-84 | 432 | 458 | 488 | 528 | 582 | 653 | 735 | 838 | 979 | 1174 | 6867 |
| 85+ | 187 | 208 | 228 | 243 | 255 | 285 | 317 | 350 | 385 | 424 | 2882 |
| Column total | 35062 | 37042 | 39149 | 41398 | 43856 | 46562 | 49577 | 52947 | 56793 | 61306 |  |
